# Plant Alkaloid Tetrandrine Is a Nuclear Receptor 4A1 Antagonist and Inhibits Panc-1 Cell Growth In Vitro and In Vivo

**DOI:** 10.3390/ijms23095280

**Published:** 2022-05-09

**Authors:** Hyo-Seon Lee, Dae Hwan Kim, In-Seon Lee, Ji-Hyun Park, Gregory Martin, Stephen Safe, Keuk-Jun Kim, Joung-Hee Kim, Byung Ik Jang, Syng-Ook Lee

**Affiliations:** 1 Department of Food Science and Technology, Keimyung University, Daegu 42601, Korea; hing1035@naver.com (H.-S.L.); inseon@kmu.ac.kr (I.-S.L.); wlgus1056@gmail.com (J.-H.P.); 2 Department of Laboratory Animal Research Support Team, Yeungnam University Medical Center, Daegu 42415, Korea; ikorando5@hanmail.net; 3 Department of Veterinary Physiology and Pharmacology, Texas A&M University, College Station, TX 77843-4466, USA; gmartin@cvm.tamu.edu (G.M.); ssafe@cvm.tamu.edu (S.S.); 4 Department of Biomedical Laboratory Science, Daekyeung College, Gyeongsan 38547, Korea; biomed@tk.ac.kr (K.-J.K.); k9j1h@naver.com (J.-H.K.); 5 Department of Internal Medicine, Yeungnam University College of Medicine, Daegu 42415, Korea; jbi@med.yu.ac.kr

**Keywords:** antagonist, apoptosis, NR4A1, pancreatic cancer, tetrandrine

## Abstract

The orphan nuclear receptor 4A1 (NR4A1) is highly expressed in human pancreatic cancer cells and exerts pro-oncogenic activity. In a previous study, we demonstrated that fangchinoline (FCN), a natural inhibitor of nuclear NR4A1, induces NR4A1-dependent apoptosis in human pancreatic cancer cells. In this study, we evaluated FCN and its structural analogs (berbamine, isotetrandrine, tetrandrine, and tubocurarine) for their inhibitory effects on NR4A1 transactivity, and confirmed that tetrandrine (TTD) showed the highest inhibitory effect in pancreatic cancer cells. Moreover, in a tryptophan fluorescence quenching assay, TTD directly bound to the ligand binding domain (LBD) of NR4A1 with a K_D_ value of 10.60 μM. Treatment with TTD decreased proliferation and induced apoptosis in Panc-1 human pancreatic cancer cells in part through the reduced expression of the Sp1-dependent anti-apoptotic gene survivin and induction of ROS-mediated endoplasmic reticulum stress, which are the well-known NR4A1-dependent proapoptotic pathways. Furthermore, at a dose of 25 mg/kg/day, TTD reduced tumor growth in an athymic nude mouse xenograft model bearing Panc-1 cells. These data show that TTD is an NR4A1 antagonist and that modulation of the NR4A1-mediated pro-survival pathways is involved in the antitumor effects of TTD.

## 1. Introduction

NR4A1, also known as TR3 or Nur77, is a member of the NR4A orphan receptor subfamily and it is induced by multiple stimuli and plays important roles in the central nervous system, inflammation, and metabolic processes [1,2]. In blood-derived cancers, NR4A1 is a tumor suppressor, whereas in solid tumors NR4A1 exhibits pro-oncogenic activities [3,4,5]. NR4A1 is highly expressed in various human tumors and is known to regulate pathways and genes associated with cancer cell proliferation, survival, migration, and invasion [4,6,7,8]. Previous studies have shown that the pro-oncogenic activity of NR4A1 in cancer cells also involves the regulation of some Sp1-mediated expression of antiapoptotic genes, such as survivin and bcl-2, and maintenance of appropriate intracellular levels of reactive oxygen species (ROS) [6,9].

Furthermore, the inhibition of NR4A1 decreases cell growth and induces apoptosis in many cancer cells in vitro and in vivo, and NR4A1 inhibitors or antagonists are being characterized as a new class of antitumor agents for the treatment of solid tumors overexpressing this receptor [4,10,11]. For example, 1,1-bis(3′-indolyl)-1-(p-hydroxyphenyl)methane (DIM-C-pPhOH) was identified as an NR4A1 antagonist, and DIM-C-pPhOH and several 3,5-disubstituted phenyl analogs inhibit cell proliferation and migration/invasion by modulating the expression of NR4A1-regulated genes in pancreatic and other cancer cell lines [4]. Moreover, many of the 3,5-disubstituted analogs of DIM-C-pPhOH inhibit tumor growth in mouse xenograft models at doses below 5 mg/kg per day.

In a recent study, this laboratory identified fangchinoline (FCN), a plant-derived NR4A1 inactivator, and showed its NR4A1-dependent anticancer activities in various cancer cell lines [10]. FCN, a bis-benzyltetrahydroisoquinoline alkaloid, and many of its structural analogs have previously been demonstrated to block the L-type Ca^2+^ channel [12], and the anticancer activity of FCN has also been reported in a variety of human cancers such as lung [13], prostate [14], and pancreas [10]. Moreover, bis(benzylisoquinoline) analogs of FCN such as tetrandrine (TTD) and berbamine (BBM) also exhibit anticancer activity via the activation of multiple proapoptotic pathways [15,16], Therefore, we hypothesized that structural analogs of FCN may exhibit anticancer activity by acting as an antagonist. This study shows for the first time that TTD, which exhibited the highest inhibition of NR4A1-dependent activity among FCN analogs, directly binds to the LBD of NR4A1 and antagonizes NR4A1-mediated transactivation in cancer cells. TTD inhibited NR4A1-regulated antiapoptotic genes and pathways in pancreatic cancer cells and also reduced tumor growth in a xenograft mouse model of pancreatic cancer. 

## 2. Results

### 2.1. TTD Is a NR4A1 Antagonist and Decreases Panc-1 Cell Growth 

**Figure 1:**
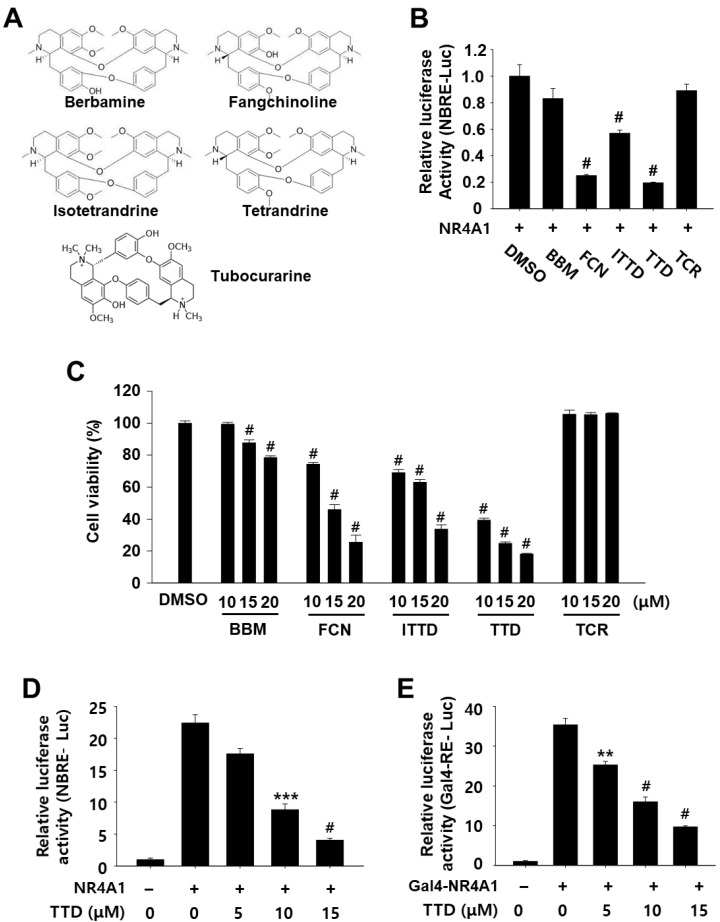
Effects of FCN and its analogs on NR4A1-dependent transactivation and cell proliferation in Panc-1 cells. (**A**) Chemical structure of FCN and its analogs. (**B**,**D**) Cells were cotransfected with NBRE-Luc (25 ng) and Flag-NR4A1 (12.5 ng) for 5 h, and then treated with each compound (20 μM) for 18 h. (**C**) Cells were treated with various concentrations of each compound for 24 h, and cell viability was determined as described in the Materials and Methods section. (**E**) Cells were cotransfected with Gal4-RE-Luc (25 ng) and 5 ng of Gal4-NR4A1 (wild type) for 5 h, and then treated with TTD for 18 h. Luciferase activity (relative to β-galactosidase) was determined, and the corresponding empty vector was used as a control. The results are presented as means ± SEM (*n* ≥ 3 replicates). ** *p* < 0.01, *** *p* < 0.005 and # *p* < 0.001 vs. DMSO, DMSO + NBRE-Luc, or DMSO + Gal4-NR4A1.

The inhibitory effect of FCN and its structural analogs, berbamine (BBM), isotetrandrine (ITTD), TTD, and tubocurarine (TCR) on NR4A1-dependent transactivation was determined in Panc-1 human pancreatic cancer cells transfected with a luciferase reporter gene containing NR4A1-binding response elements (NBRE-Luc) (Figure 1A,B). FCN, ITTD, and TTD at 15 µM significantly inhibited luciferase activity in Panc-1 cells transfected with NBRE-luc, whereas BBM and TCR at the same concentration showed no significant effect (Figure 1B). In addition, we investigated the effects of these compounds on Panc-1 cell viability, and the results in Figure 1C showed that FCN, ITTD, and TTD significantly inhibited cell growth in a dose-dependent manner, but BBM and TCR showed weak or no inhibitory effects on cell viability. TTD which inhibited NR4A1-dependent transactivation was a more potent inhibitor of cell growth than other analogs at all concentrations, and after 24 h treatment, the growth-inhibitory IC_50_ (the half maximal inhibitory concentration) value of FCN, ITTD, and TTD was 14.30, 16.53, and 10.20 µM, respectively. We further confirmed the dose-dependent effects of TTD on NR4A1-dependent transactivation using two different systems, the NBRE reporter gene and the Gal4-NR4A1 fusion construct (Gal4-NR4A1), with a Gal4 reporter gene containing Gal4 response elements (Gal4-RE-Luc). Treatment with TTD (5–15 µM) significantly inhibited NBRE reporter gene activity in a dose-dependent manner in Panc-1 (Figure 1D) cells. TTD also dose-dependently decreased luciferase activity in cells transfected with Gal4-RE-Luc and Gal4-NR4A1 (Figure 1E). 

We also investigated the effects of TTD on NR4A1 protein expression in Panc-1 cells. Western blot data showed that TTD did not affect protein expression of NR4A1 in Panc-1 cells (Supplemental Figure S1), suggesting that there is no relationship between TTD-mediated inhibition of NR4A1-dependent transactivation and regulation in NR4A1 expression.

**Figure 2:**
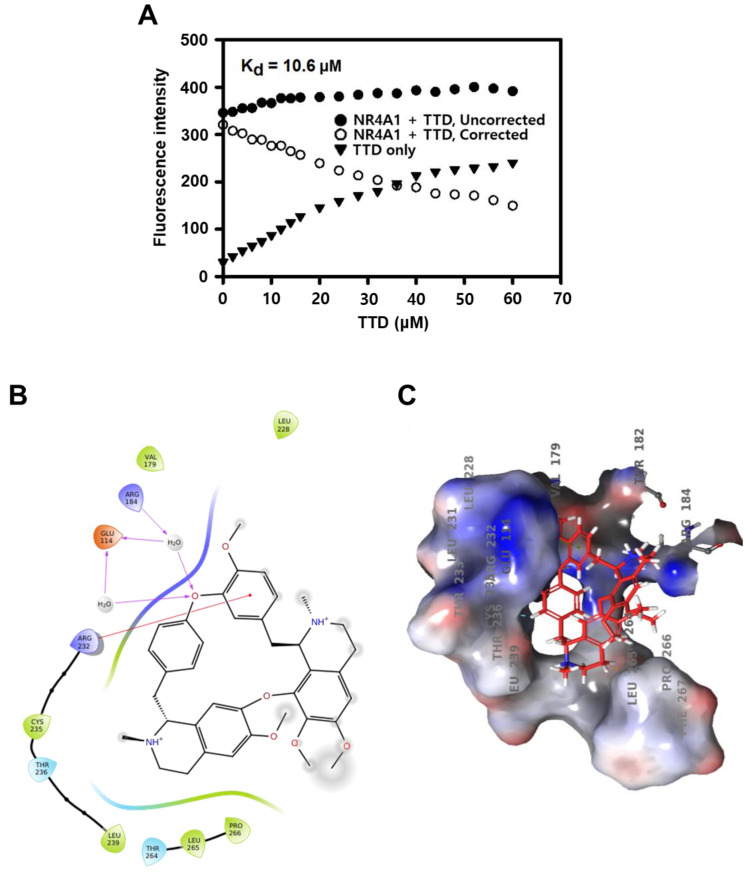
TTD binds NR4A1. (**A**) Direct binding of TTD with the LBD of NR4A1 was determined by the fluorescence quenching of a Trp residue in the LBD. Example of TTD interactions with amino acid side chains (**B**) and the binding pocket of NR4A1 (**C**).

Moreover, we investigated the interaction of TTD with NR4A1 by determining the direct binding of TTD to the LBD of NR4A1 and subsequent Trp fluorescence quenching. The binding curves for TTD showed that TTD bound to NR4A1 LBD and its K_D_ value was 10.60 µM (Figure 2A). These results suggest that TTD directly binds the LBD of NR4A1 and antagonized NR4A1-dependent transactivation.

TTD is bis-benzyl isoquinoline alkaloid derivative and contains N-methyl group substituents at the 2 and 2′ positions and methoxy group substituents at positions 6, 6′, 7, and 12. Docking and modeling TTD to the LBD of NR4A1 using the Schrodinger Maestro software resulted in unique binding orientations for TTD within the ligand-binding pocket of the NR4A1 LBD (Figure 2B). TTD was situated on the surface of the ligand binding pocket with the hydroxy and methoxy groups exposed to solvent. The docking model of TTD predicted a pi-cation interaction between Arg 232 (Arg 563 in full-length NR4A1) and the 6′-methoxy phenyl ring and calculation of its predicted binding energy yielded −3.3 kcal/mol (Figure 2C). The predicted docking score for TTD was in agreement with the binding affinity results from the direct binding analysis. 

**Figure 3:**
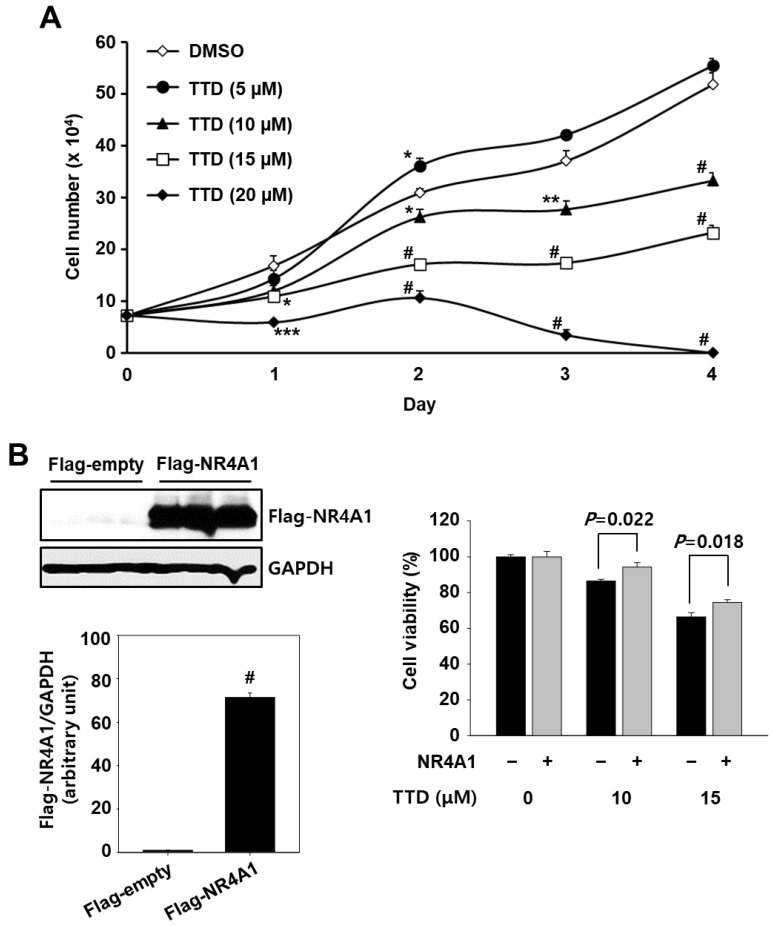
TTD inhibits cell proliferation in pancreatic cancer cells. (**A**) Panc-1 cells were treated with various concentrations of TTD for the indicated time. (**B**) NR4A1-mediated cell growth inhibition by TTD. Cells were transfected with either Flag-empty or Flag-NR4A1 for 6 h and at 24 h after transfection, cells were treated with TTD for 24 h. Whole cell lysates were analyzed by western blot analysis. GAPDH was used as a loading control. Cell viability was determined as described in the Materials and Methods section. The results are presented as means ± SEM (*n* ≥ three replicates). * *p* < 0.05, ** *p* < 0.01, *** *p* < 0.005 and # *p* < 0.001 vs. DMSO or Flag-empty.

Previous studies have shown that NR4A1 silencing and that the NR4A1 antagonist reduced cell proliferation and induced apoptosis in many different cancer cell lines, including Panc-1 cells [4,10,11]. Here, we also observed that TTD significantly inhibited the proliferation of Panc-1 cells (Figure 3A and Supplemental Figure S2), and its growth-inhibitory IC_50_ values for 24 and 48 h treatments were 19.33 and 12.94 µM, respectively. Further investigation into whether TTD acts by antagonizing NR4A1 was performed in a rescue experiment and confirmed that the growth inhibitory effect of TTD was partially but significantly reversed in Panc-1 cells overexpressing NR4A1 (Figure 3B). In addition, the effect of TTD on viability of immortalized normal cell lines including HaCaT human keratinocytes, AML-12 mouse hepatocytes, and 3T3-L1 mouse embryonic fibroblasts was examined. As shown in Supplemental Figure S3, TTD (5–20 μM), except for the mild toxicity to HaCaT cells at 20 μM, did not significantly affect the viability of normal cells with very low NR4A1 expression, indicating that NR4A1-dependent survival mechanisms may not be important for these normal cells, and TTD has specific cytotoxicity to cancer cells overexpressing NR4A1.

### 2.2. TTD Induces Apoptosis through Inhibition of NR4A1-Mediated Pro-Oncogenic Pathways 

Our previous studies showed that the antiapoptotic activity of NR4A1 was due in part to the regulation of pro-survival genes with GC-rich promoters such as survivin (mechanism 1) [6] and maintenance of intracellular oxidative stress at low levels in cancer cells (mechanism 2) [9]. In addition, NR4A silencing and treatment with NR4A1 inactivators induced apoptosis in pancreatic cancer cells and tumors [4]. Thus, the effects of TTD on the NR4A1-mediated antiapoptotic pathways in Panc-1 cells were further investigated. 

**Figure 4:**
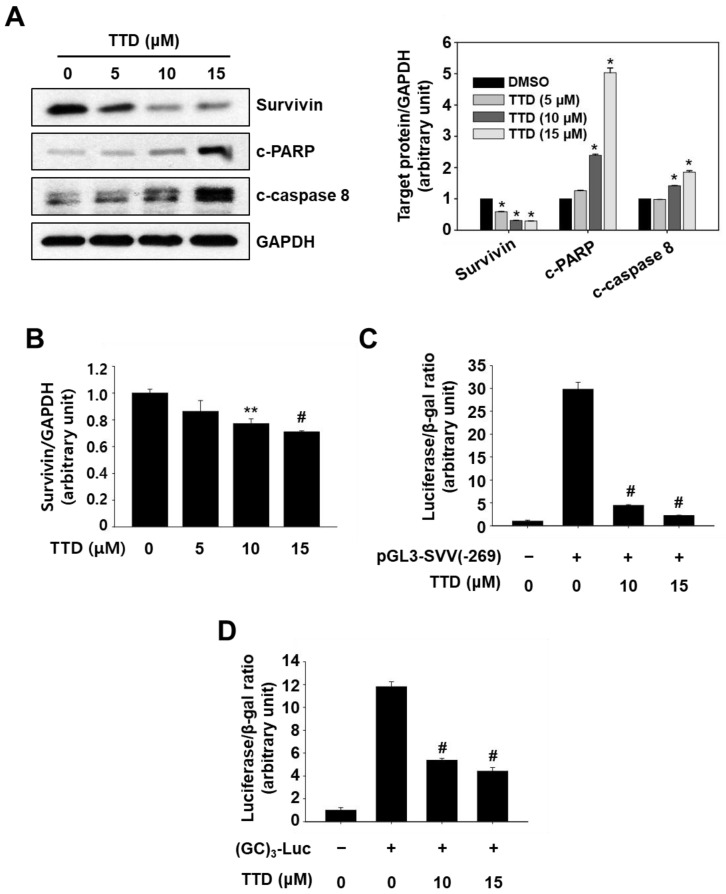
TTD inhibits Sp1-mediated survivin expression and induces apoptosis in pancreatic cancer cells. (**A**) Panc-1 cells were treated with various concentrations of TTD for 24 h, and whole-cell lysates were analyzed by western blot analysis. GAPDH was used as a loading control. The molecular weight of c-PARP and c-caspase 8 is 89 kDa and 41/43 kDa, respectively. (**B**) Cells were treated with TTD for 18 h, and the survivin mRNA level was determined by real-time PCR as described in the Materials and Methods section. (**C**) Cells were transfected with 25 ng of pGL3-SVV (-269) (**C**) or 25 ng of pGL3-(GC)_3_-Luc (**D**) for 5 h and then treated with TTD for 18 h. Luciferase activity (relative to β-galactosidase) was determined, and the corresponding empty vector was used as a control. The results are presented as means ± SEM (*n* ≥ 3 replicates). * *p* < 0.05, ** *p* < 0.01, and # *p* < 0.001 vs. DMSO.

As shown in Figure 4A, TTD increased cleavage of PARP and caspase-8 in Panc-1 cells, and these effects of TTD were similar to those observed following treatment with other NR4A1 inactivators such as DIM-C-pPhOH, FCN, and BCA [6,10,11,17]. TTD also decreased protein and mRNA expressions of survivin, a major NR4A1-regulated antiapoptotic gene (Figure 4A,B). To further examine the impact of TTD on specificity protein 1 (Sp1)-dependent modulation of survivin, a survivin promoter-reporter pGL3-SVV(−269) containing multiple GC boxes and an Sp1-regulated promoter construct harboring tandem repeats of GC-rich sequences ([GC]_3_-Luc) were used. Treatment with TTD (10 and 15 µM) dramatically reduced luciferase activity in Panc-1 cells transfected with the survivin and GC-rich promoter constructs. These results are consistent with previous results with NR4A1 antagonists indicating that NR4A1 plays a role in regulation of Sp-dependent genes that can be inhibited by NR4A1 antagonists [6,10,11]. These results also confirmed that TTD acts as an NR4A1 antagonist with respect to survivin expression. 

**Figure 5:**
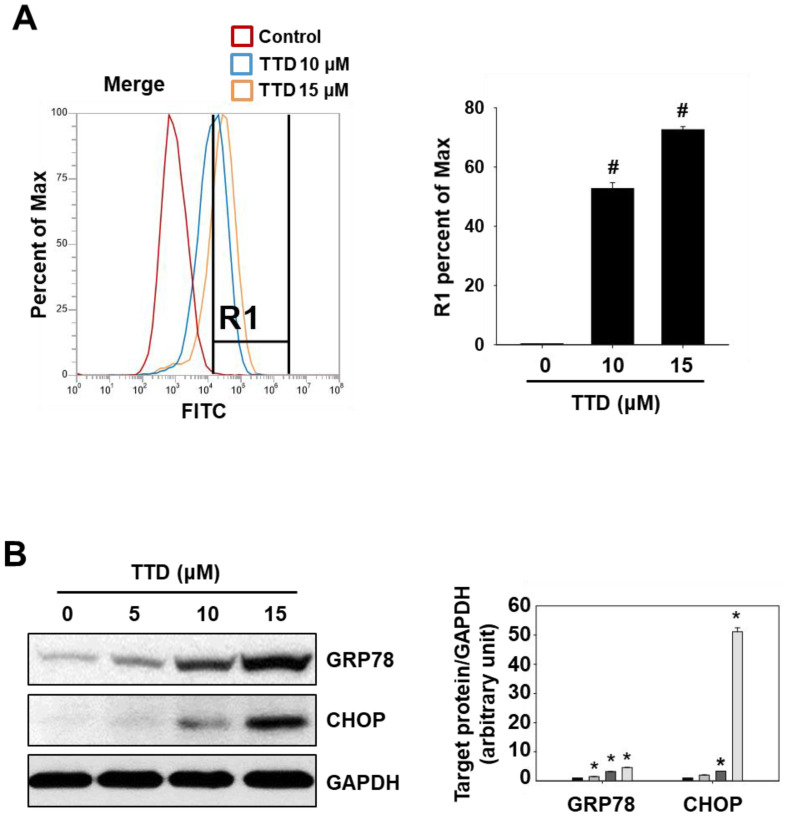
TTD increases ROS production and induces ER stress in pancreatic cancer cells. (**A**) Panc-1 cells were treated with TTD for 6 h, and the intracellular ROS level was measured by flow cytometry as described in the Materials and Methods section. The results are presented as means ± SEM (*n* ≥ 3 replicates). # *p* < 0.001 vs. DMSO. (**B**) Cells were treated with TTD for 24 h, and whole-cell lysates were analyzed by western blot analysis. GAPDH was used as a loading control. * *p* < 0.05 vs. DMSO.

NR4A1 is known to suppress ROS-mediated ER stress by maintaining low levels of oxidative stress, and previous studies also showed that DIM-C-pPhOH induced apoptosis via the ROS/ER stress-mediated pathway in pancreatic cancer cells [9]. Thus, we further examined the effects of TTD on cellular ROS levels and the induction of ER stress. Treatment with TTD (10 and 15 µM) for 6 h significantly increased intracellular ROS levels without affecting cell viability (Figure 5A and Supplemental Figure S4). TTD treatment for 24 h also induced ER stress markers, GRP78 and CCAAT/enhancer-binding protein homologous protein (CHOP) in Panc-1 cells (Figure 5B), suggesting that cellular ROS generation and induction of ER stress also contribute to apoptosis in TTD-treated cells. 

### 2.3. TTD Reduces Tumor Growth in a Xenograft Mouse Model of Panc-1 Cells

**Figure 6:**
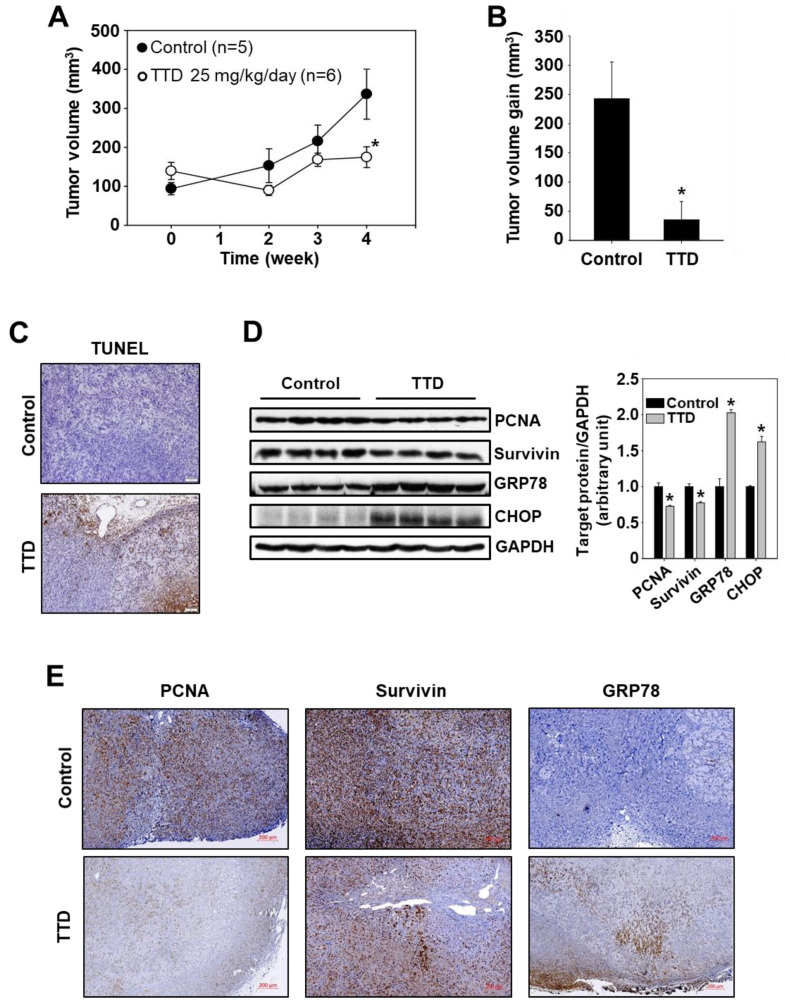
Effects of TTD on growth and apoptosis in a mouse xenograft model of human pancreatic cancer. Panc-1 cells were implanted into athymic nude mice as described in the Materials and Methods section. Each mouse was dosed by oral gavage with either corn oil (control) or TTD (25 mg/kg/day) for four weeks. (**A**,**B**) Median tumor volumes were calculated as described in the Materials and Methods section. The results are presented as means ± SD. * *p* < 0.05 vs. the control group. (**C**) Tumor sections were stained using the DeadEnd colorimetric kit as described in the Materials and Methods section. Images of the apoptotic tumor cells were collected at high (×100) magnification. (**D**) Tumor lysates were analyzed by western blot analysis, and GAPDH was used as a loading control. The results are presented as means ± SEM (*n* = 4 replicates). * *p* < 0.05 vs. the control group. (**E**) Immunohistochemistry shows the expression of PCNA, survivin, and GRP78 in tumor sections.

Antitumor activity of TTD in a xenograft mouse model of pancreatic cancer was investigated to complement the in vitro data observed for TTD in Panc-1 cells. Panc-1 cells (1 × 10^7^ cells/150 µL) in Matrigel were injected into the flanks of individual athymic nude mice, and after seven days mice were treated with TTD (25 mg/kg/day) for four weeks, and the effect on tumor growth and volume was then measured. The results in Figure 6A,B demonstrate that TTD significantly decreased tumor volumes (tumor volume at four-weeks, control vs TTD-treated mice = 337 vs. 175 mm^3^, difference = 162 mm^3^, 95% confidence interval = 122 to 227 mm^3^) compared to the tumors in the control group. At this dose, significant changes in organ weights, body, or food intake were not observed (Supplemental Tables S1 and S2), and organs were normal in size and appearance (data not shown). Moreover, no significant changes in serum levels of alkaline phosphatase (ALP), alanine aminotransferase (ALT), aspartate aminotransferase (AST), blood urea nitrogen (BUN), and creatinine and in histology of three major organs, kidney, liver, and spleen in TTD-treated mice (Supplemental Figure S5A,B) was detected, suggesting that TTD induced no discernible toxicity to the mice. TUNEL staining of tumor tissues demonstrated that TTD induced apoptosis (Figure 6C), and western blot analysis of tumor lysates showed that TTD decreased the protein level of PCNA and survivin and increased protein levels of GRP78 and CHOP (Figure 6D). The expression of survivin and GRP78 was also decreased and increased, respectively, in the tumor tissues of TTD-treated mice compared to the control group (Figure 6E), complementing the results of the in vitro studies. The comparable effects observed in vivo and in vitro demonstrated that TTD is an NR4A1 antagonist that acts as an antagonist to inhibit NR4A1-dependent pro-oncogenic pathways, and this confirms a potential clinical role for TTD as another NR4A1 antagonist in pancreatic cancer chemotherapy.

## 3. Discussion

Various types of antitumor agents regulate apoptosis in cancer cells through NR4A1-mediated genomic and nongenomic pathways, and earlier studies of NR4A1 mainly focused on the nongenomic mechanisms of NR4A1 [18,19]. However, since two novel pro-survival mechanisms of NR4A1 in pancreatic cancer cells have been identified [6,9], many studies have focused on the genomic functions of NR4A1 in cancer [4]. Results of the gene knockdown studies demonstrated that the pro-oncogenic activity of nuclear NR4A1 involves the regulation of the antiapoptotic gene survivin through the formation of an NR4A1/p300/Sp1 complex bound to its GC-rich survivin promoter sequences and the regulation of ROS-mediated ER stress [6,9]. Recently, a series of bis-indole-derived analogs (CDIMs) that bind to the LBD of NR4A1 and act as NR4A1 antagonists have been developed, and treatment with several CDIMs have been shown to inhibit the NR4A1-regulated pro-oncogenic responses in multiple cancer cells including pancreatic cancer [4].

Our previous research has identified FCN as an inactivator of NR4A1-mediated responses and showed that treatment of pancreatic cancer cells with FCN was highly effective at inhibiting cell growth and survival [10]. The efficacy of FCN was due in part to its suppression of the antiapoptotic gene survivin and the induction of ER stress-mediated apoptosis. In the present study, based on the results illustrated in Figure 1 demonstrating that TTD is the most potent inhibitor of nuclear NR4A1 among the structural derivatives of FCN, we hypothesized that TTD may be an NR4A1 antagonist to inhibit cancer cell growth in both cell culture and in vivo models. 

Results from the fluorescence quenching assay (Figure 2) confirm that TTD directly binds to the LBD of NR4A1, and it also inhibits two NR4A1-regulated (dependent) pro-survival pathways, namely an Sp1-mediated regulation of survivin (Figure 4) and a regulation of ROS-mediated ER stress (Figure 5) in Panc-1 cells. Furthermore, as shown in Supplemental Figure S6, TTD (≥10 µM) significantly inhibited NBRE reporter gene activity and cell proliferation (IC_50_ value = 15.89 µM) in a dose-dependent manner in MiaPaCa-2 cells, another human pancreatic cancer cell line known to overexpress NR4A1 [11]. Treatment with TTD in MiaPaCa-2 cells also decreased survivin expression, increased expression of ER stress marker proteins, and induced apoptosis, confirming that TTD inhibited cell proliferation in various types of pancreatic cancer cells.

We also observed that TTD at a dose of 25 mg/kg/day was a potent inhibitor of pancreatic tumor growth in athymic nude mice bearing Panc-1 cells (Figure 6). Complementary data from in vitro and in vivo studies show for the first time that TTD is an NR4A1 antagonist that acts as an antagonist in Panc-1 cells. Moreover, no biochemical or histological signs of general toxicity were observed in mice treated with TTD for four weeks, and no other significant toxic effects were observed in reference to organ weight, body weight, and clinical signs in TTD-treated mice (Supplemental Figure S5; Supplemental Tables S1 and S2). TTD is a natural compound that belongs to the bis-benzylisoquinoline alkaloid family and numerous investigations have demonstrated that TTD possesses diverse pharmacological activities such as anti-inflammatory [20], antifibrotic [21], antimicrobial [22], and neuroprotective activities [23]. Recently, anticancer properties of TTD in both cell culture and animal models have also been demonstrated in several cancers, including breast [24], cervical [25], lung [26], oral [27], and pancreas [24,28,29]. The anticancer mechanisms of TTD include the induction of apoptosis via VEGF/HIF-1α/ICAM-1 signaling pathway, the induction of autophagy, and cell cycle regulation [26,27,28]. In Panc-1 cells with p53 mutation (R273H), TTD has also been shown to inhibit cell growth and survival through the ROS-mediated induction of apoptosis and the regulation of the mTOR pathway [24,29]. The former anticancer mechanism of TTD is consistent with the present study and is likely NR4A1-dependent; however, the latter mechanism seems to be an NR4A1-independent anticancer mechanism of TTD since NR4A1 is known to regulate the AMPKα/mTOR signaling pathway only in p53 wild-type cancer cells [7], indicating that NR4A1-dependent anticancer activity does not fully cover TTD-mediated anticancer mechanisms, but other pathways are also involved, as previously reported.

Approximately 80% of anticancer drugs approved by the US FDA over the past 30 years have been found to be natural products, their derivatives, or drugs synthesized based on the structure of natural products [30]. Moreover, approximately 14% of all US FDA-approved small-molecule drugs target nuclear receptors due to their critical roles in maintaining homeostasis and their role in pathophysiology, and numerous studies are being conducted to discover small molecules that target nuclear receptors including NR4A1 from natural products. This study confirms for the first time that TTD, a bis-benzylisoquinoline alkaloid, is an NR4A1 antagonist that antagonizes NR4A1-mediated pro-oncogenic activity, and we are currently examining structurally-related alkaloids and other natural products for their activities as NR4A1 antagonists. 

## 4. Materials and Methods

### 4.1. Cell Lines and Plasmids

All cell lines were obtained from Korean Cell Line Bank and maintained as previously described [9,10]. Panc-1 and MiaPaCa-2 cells were maintained in Dulbecco’s Modified Eagle Medium supplemented with 10% fetal bovine serum (Welgene, Gyeongbuk, Korea) at 37 °C in a humidified CO_2_ incubator (5% CO_2_). All the constructs used in this study have been previously described [7,10].

### 4.2. Antibodies, Reagents, Quantitative Real-Time PCR, and Western Blot Analysis

All antibodies except for NR4A1 and the 78-kDa glucose-regulated protein (GRP78) were purchased from Cell Signaling Technology (Beverly, MA, USA). NR4A1 and GRP78 antibodies were obtained from Abcam (Cambridge, MA, USA) and Santa Cruz Biotechnology (Santa Cruz, CA, USA), respectively. Luciferase reagent and β-galactosidase (β-Gal) reagent were purchased from Promega (Madison, WI, USA). FCN and its structural analogs were purchased from Toronto Research Chemicals (Toronto, ON, Canada). Western blot and quantitative real-time PCR (qPCR) analyses were performed as previously described [31]. For western blotting, cellular lysates were prepared using a RIPA lysis buffer containing protease and phosphatase inhibitors, and protein concentration was determined using the Bio-Rad protein assay. Aliquots (30 µg/lane) of cellular proteins were electrophoresed on 10% or 12% SDS–polyacrylamide gel electrophoresis (PAGE) and transferred to an Immobilon-P-membrane (Millipore, Burlington, MA, USA). The membrane was allowed to react with a specific antibody and detection of specific proteins was performed by enhanced chemiluminescence. For qPCR analysis, total RNA was extracted using TRIzol reagent (Ambion Inc., Carlsbad, CA, USA) according to the manufacturer’s instructions. cDNA was prepared from the total RNA using Reverse Transcription System (Takara Bio Inc., Shiga, Japan). Each PCR was carried out in triplicate in a 20-μL volume using SYBR Green Premix (Takara Bio Inc., Shiga, Japan) for 15 min at 95 °C for initial denaturing followed by 40 cycles of 95 °C for 30 s and 60 °C for 1 min in the Takara Thermal Cycler Dice Real Time System. The Takara Dissociation Curves software was used following a brief thermal protocol (95 °C for 15 s and 60 °C for 15 s followed by a slow ramp to 95 °C) in each PCR amplification. The sequences of the primers used for real-time PCR were as follows: survivin sense 5′-CAG ATT TGA ATC GCG GGA CCC-3′, antisense 5′-CCA AGT CTG GCT CGT TCT CAG-3′ and GAPDH sense 5′-GTA TGA CTC CAC TCA CGG CA-3′, antisense 5′-GGT CTC GCT CCT GGA AGA TG-3′. 

### 4.3. Cell Proliferation Assay, Transfection, and Reporter Gene Assay

The MTT (3-(4,5-dimethylthiazol-2-yl)-2,5-diphenyltetrazolium bromide) assay was used to assess cell viability as described previously [11]. Briefly, cells were plated in 48-well culture plate at a density of 3 × 10^4^ cells/well in DMEM culture medium and allowed to attach for 18 h. After incubation, the medium was discarded and replaced with 200 µL of new medium containing various concentrations of TTD. After 24 h of culture, 20 µL MTT solution (2.5 mg/mL) was added to each well. The insoluble formazan crystals were dissolved with DMSO after 4 h of incubation with MTT solution, and the optical density was read at 550 nm using a microplate reader. For luciferase reporter assays, Panc-1 cells were plated in a 48-well plate (3 × 10^4^ cells/well) and grown for 18 h. Cells were then co-transfected with each reporter construct (25 ng) and the β-Gal expression construct (5 ng) for 4 h using Lipofectamine 2000 reagent (Invitrogen, San Diego, CA, USA). Then, the cells were treated with each compound for 18 h, and luciferase activity was measured and normalized with the β-Gal activity.

### 4.4. Direct Binding Assay

The quenching of NR4A1 tryptophan (Trp) fluorescence by adding a ligand was obtained essentially as described previously (Lee et al., 2014b); the ligand-binding domain (LBD) of NR4A1 (0.5 µM) was incubated with various concentrations of TTD and fluorescence was obtained under 285 nm excitation and 300–420 nm emission range (each slit width = 5 nm). The ligand-binding affinity (Kd) was determined by measuring the dose-dependent NR4A1 Trp fluorescence intensity at 330 nm emission.

### 4.5. Computation-Based Molecular Modeling

Molecular modeling was carried out using Schrödinger software on the Maestro modeling platform (Schrödinger Release 2020-1, Schrödinger, LLC, New York, NY, USA, 2020) as previously described [17]. The crystal structure was prepared using the Maestro Protein Preparation Wizard according to the crystal structure coordinates for the NR4A1 LBD downloaded from the Protein Data Bank (https://www.rcsb.org; accessed on 14 August 2021), and a three-dimensional structure of TTD was prepared using the OPLS3e force field in the LigPrep panel in Maestro. Glide was utilized to dock TTD to the NR4A1 LBD, predict the lowest energy ligand-binding orientation, and calculate the predicted binding energy in units of kcal/mol.

### 4.6. Measurement of Intracellular Level of ROS

The cellular ROS level was measured using the 2′7′-dichlorofluorescin diacetate (DCF-DA) fluorescent probe as previously described [10]. In brief, Panc-1 cells were seeded in 6-well plates (3.5 × 10^6^ cells/well) and treated with TTD for 6 h. Cells were then incubated with DCF-DA (25 μM in PBS) for 30 min and detached with trypsin-EDTA. The cells were resuspended in PBS, and cellular fluorescence intensity was measured by flow cytometric analysis (BD FACSVerse, BD-Biosciences, San Jose, CA, USA). 

### 4.7. Animals and Experimental Design

Male athymic nude mice (NCr-nu/nu; 5-week-old) were purchased from Koatech Inc. (Pyeongtaek-si, Korea). Animals were housed in a climate-controlled environment (21 °C ± 5 % humidity) with a 12-h light/dark cycle and allowed to acclimatize to the facility for seven days. The mice were allowed free access to water and a control (10 kcal% fat) pellet diet (Cat. No. D10001; Research Diets, Inc., New Brunswick, NJ, USA). Panc-1 cells were harvested as single cells and injected into the flanking region as previously described [32]. One week after cell injection, TTD (25 mg/kg/day in corn oil, *n* = 6) or corn oil (*n* = 5) was orally administered to tumor-bearing mice for four weeks. Tumor volume was measured weekly and on the day of sacrifice, body weight and organ weight were also measured. Tumor volume was calculated by using the (width) 2 × (length) × 0.5 formula. Then, the mice were sacrificed under anesthesia for 24 h after four weeks of treatment, and their serum and tissues were aseptically removed. The serum was obtained by centrifuging at 4 °C (3000 rpm for 15 min) and then immediately subjected to biochemical analyses. For histological examination, the liver, kidney, spleen, and tumor from each animal were fixed in 10 % formalin, embedded in paraffin, processed into 3-μm thick sections, and subjected to hematoxylin and eosin staining. All animals were maintained and used in accordance with the guidelines of the Institutional Animal Care and Use Committee of the College of Medicine, Yeungnam University (YUMC-AEC2016-029).

### 4.8. Immunohistochemical Analysis

Immunohistochemistry (IHC) was carried out using DAKO Envision Polymer techniques (DAKO, Hamburg, Germany) according to the manufacturer’s instructions. In brief, tissue sections were deparaffinized and rehydrated, and heat-induced antigen retrieval was then performed using a citrate buffer (pH 6.0) for 45 min in a microwave oven. The endogenous peroxidase activity was quenched with H_2_O_2_ (3% in MeOH) for at least 10 min. Tissue sections were then incubated for 60 min with a rabbit PCNA polyclonal antibody (Abcam, 1:50), a rabbit BiP (C50B12) monoclonal antibody (Cell Signaling, 1:100), and a rabbit survivin (71G4B7) monoclonal antibody (Cell Signaling, 1:200) at 25 °C in a humidified chamber. After 3 washes of 5 min with TBS, tissue sections were incubated for color development sequentially with HRP-conjugated anti-rabbit IgG and 3,3′-diaminobenzidine/H_2_O_2_. The sections were then counterstained with hematoxylin, dehydrated, and mounted. IHC images were acquired using an Aperio CS2 slide scanner (Leica Biosystems, Nussloch, Germany) and processed with Aperio ImageScope software. 

### 4.9. Statistical Analysis

Statistical analysis was performed using a Student’ s *t*-test with Sigma Plot 10.0 (Systat Software Inc., San Jose, CA, USA). The results are presented as means ± SEM (*n* ≥ 3), and *p*-values below 0.05 were considered to be significant. 

## 5. Conclusions

In conclusion, TTD is a natural ligand of NR4A1 that acts as an antagonist, and treatment with TTD inhibited pancreatic cancer cell growth in both cell culture and in vivo studies in part through the regulation of two NR4A1-dependent apoptotic pathways. The results in the present study enhance our understanding of the antitumor mechanisms of TTD and suggest that TTD can be used alone or in drug combinations for targeted therapy for cancer patients overexpressing NR4A1.

## Supplementary Materials

The following supporting information can be downloaded at: https://www.mdpi.com/article/10.3390/ijms23095280/s1.
