# Supplementary material for: Plant Alkaloid Tetrandrine Is a Nuclear Receptor 4A1 Antagonist and Inhibits Panc-1 Cell Growth In Vitro and In Vivo
Source: Int J Mol Sci. 2022 May 9;23(9):5280. doi: 10.3390/ijms23095280 (PMC9104798; doi:10.3390/ijms23095280)
Supplement: Supplementary file 1 [file ijms-23-05280-s001.zip › ijms-1712932-supplementary.pdf]

SUPPLEMENTAL METERIALS

2 Tables

6 Figures

Supplemental Table S1. Effects of TTD on body weight and food intake in a mouse xenograft model of human pancreatic cancer

	Body weight gain (g/4 weeks)	Food intake (g/week/mouse)
Control	2.50±0.00	21.25±5.71
TTD (25 mg/kg/day)	2.08±1.46	20.45±4.68

All results are presented as means ± SD (n=5 or 6).

Supplemental Table S2. Effects of TTD on organ weights in a mouse xenograft model of human pancreatic cancer

	Organ weight (mg/g body weight)				
	Liver	Spleen	Kidney	Heart	Lung
Control	47.51±1.93	5.01±0.47	13.33±0.80	4.75±0.52	5.25±0.41
TTD (25 mg/kg/day)	41.77±3.50	4.95±0.61	14.16±1.00	4.88±0.24	5.21±0.29

All results are presented as means ± SD (n=5 or 6).

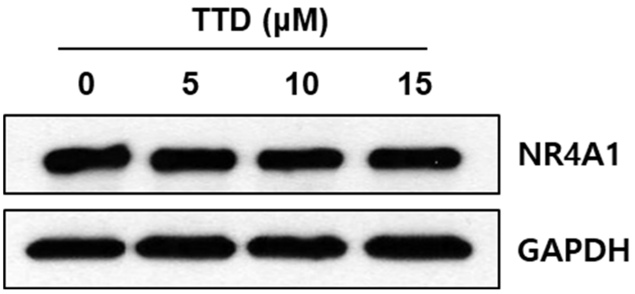

Supplemental Figure S1. Effects of TTD on protein expression of NR4A1 in Panc-1 cells. Cells were treated with TTD for 24 h, and whole cell lysates were analyzed by western blot analysis. GAPDH was used as a loading control.

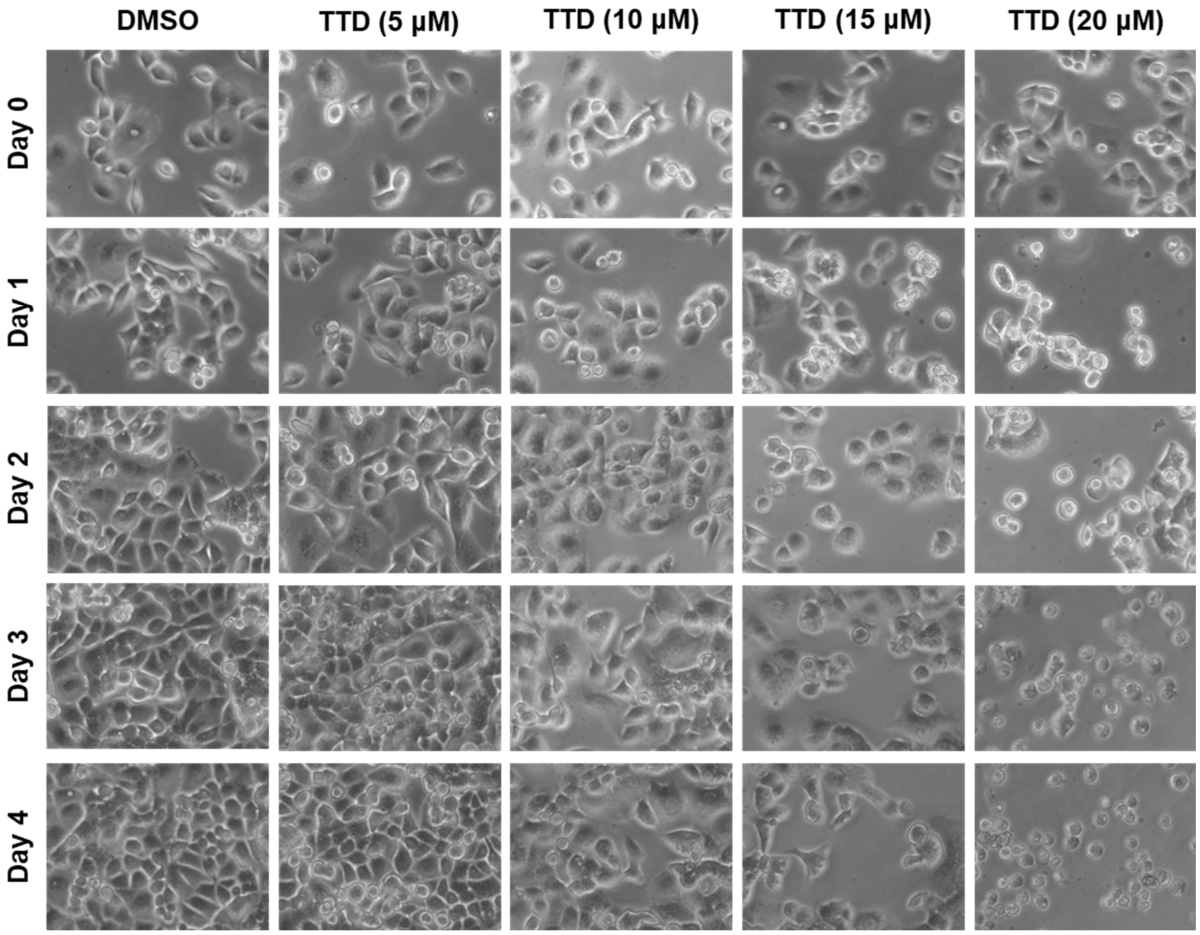

Supplemental Figure S2. Effect of TTD on the morphology of Panc-1 cells. Cells were treated with TTD for 1, 2, 3, or 4 days, and cells in each well were photographed using a phase-contrast microscope at x100 magnification. Representative images are shown.

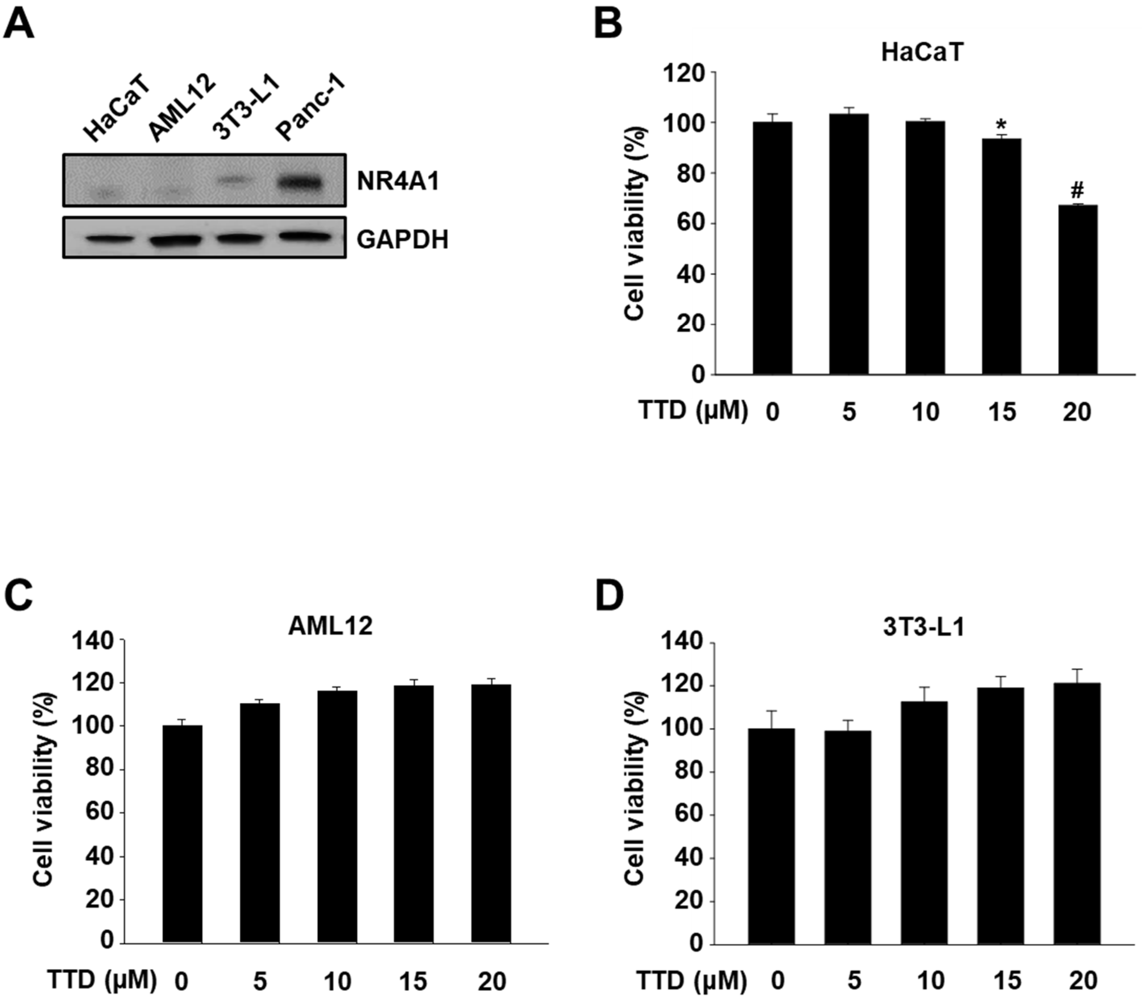

Supplemental Figure S3. Effects of TTD on viability of normal cell lines. (A) Whole-cell lysates from each cell lines were analyzed by western blot analysis, and GAPDH was used as a loading control. (B-D) Cells (3×10^4 cells/well) were plated in 48-well plate and grown overnight. Cells were then treated with various concentrations of TTD for 24 h, and cell viability was determined.

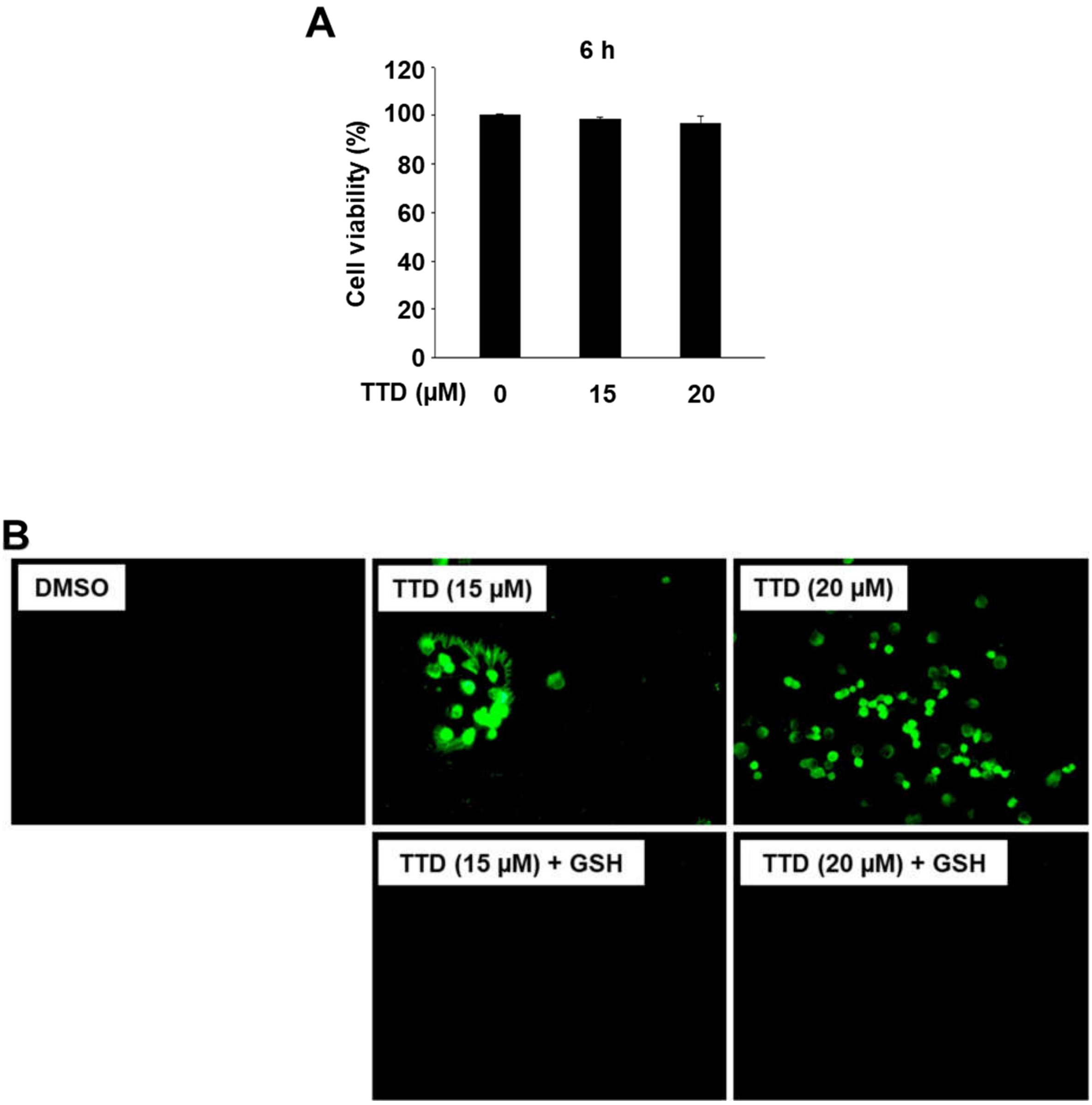

Supplemental Figure S4. TTD increases ROS production in Panc-1 cells. Cells were treated with TTD in the presence or absence of GSH (10 mM) for 6 h, and intracellular ROS level was measured by fluorescence microscopy (x200) as described in the Materials and methods.

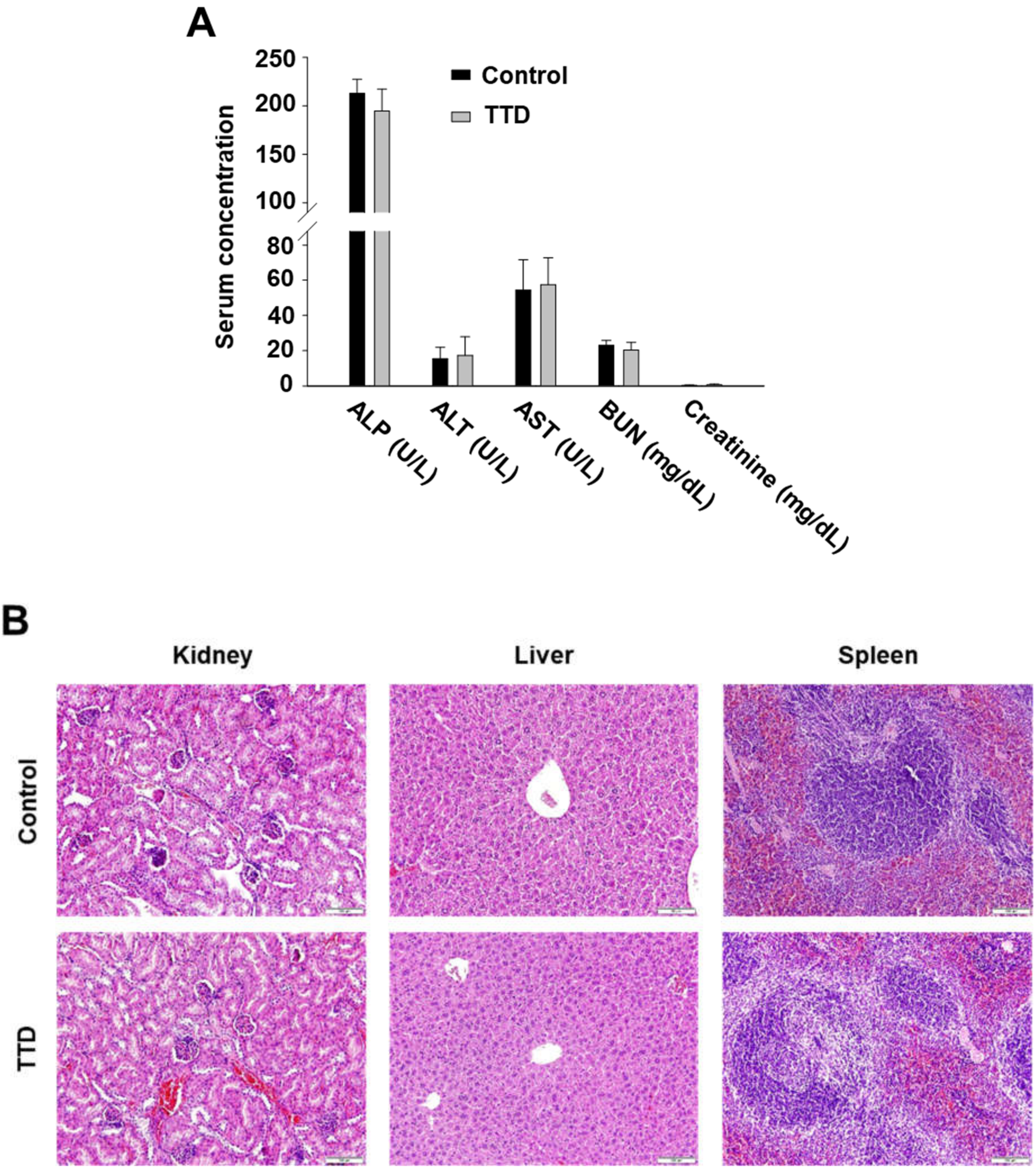

Supplemental Figure S5. Effects of TTD on serum levels of liver and kidney biomarkers and histology of three major organs in a mouse xenograft model of human pancreatic cancer. Serum samples (A) and tissue sections (B) were prepared and analyzed as described in the Materials and Methods. The results are presented as means ± SD, and images were collected at high (x 200) magnification.

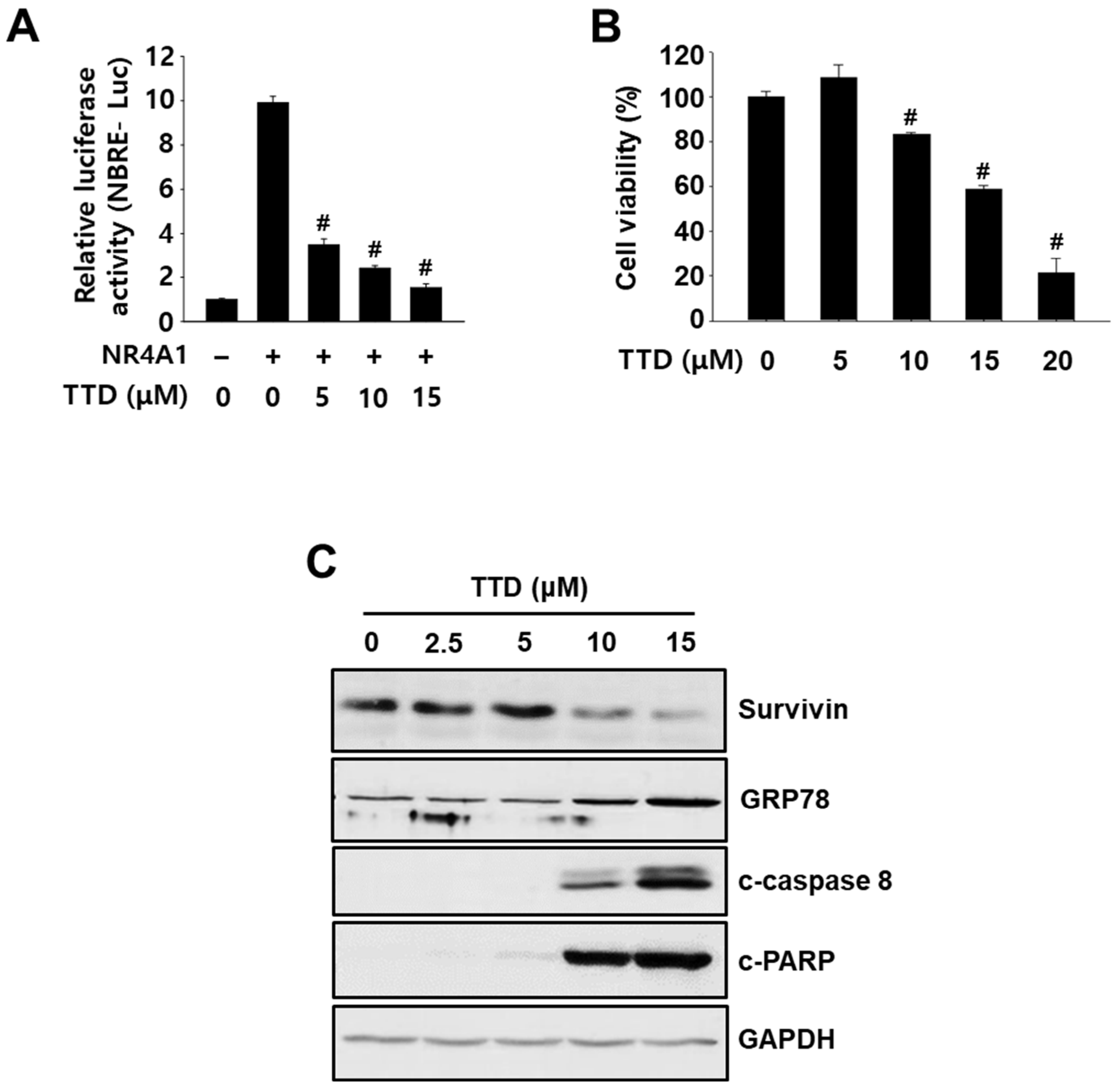

Supplemental Figure S6. TTD inhibits NR4A1-dependent transactivation and induces apoptosis in MiaPaCa-2 cells. (A) Cells were cotransfected with NBRE-Luc (25 ng) and Flag-NR4A1 (12.5 ng) for 5 h, and then treated with TTD for 18 h. Luciferase activity (relative to β-galactosidase) was determined, and the corresponding empty vector was used as a control. (B) Cells were treated with TTD for 24 h, and cell viability was determined. (C) Cells were treated with TTD for 24 h, and whole-cell lysates were analyzed by western blot analysis.

GAPDH was used as a loading control. The results are presented as means ± SEM (n≥3).

$^{\#}$*P*<0.001 vs. DMSO + NBRE-Luc or DMSO.
